# Black is the new blue? A cross-sectional Gulf Cooperation Council (GCC) survey of colour preferences for scrubs, PPE and the dental clinical environment

**DOI:** 10.1038/s41405-026-00426-z

**Published:** 2026-04-13

**Authors:** Abubaker Qutieshat, Reem T. Al Amry, Zahra Mustafa, Mahmoud Rezk, Ghaid L. Al Riyami, Majid Hamid-Oudjana, Laila Kebab, Ahmed Al Wahaibi, Rayhana Aouididi, Klara Avetisyan

**Affiliations:** 1 Department of Restorative Dentistry, College of Dental Medicine, University of Sharjah, Sharjah, United Arab Emirates; 2 Oman Dental College, Muscat, Oman

**Keywords:** Dental equipment, Dental business

## Abstract

**Background:**

Colour cues in dental clinics may shape perceptions of professionalism, comfort, hygiene and anxiety, yet evidence from Gulf Cooperation Council (GCC) settings across attire, PPE and the operatory environment remains limited. This study examined colour preferences across Oman, the United Arab Emirates (UAE) and Bahrain.

**Methods:**

A cross-sectional, web-based survey was completed by non-healthcare university students in Oman (*n* = 193), UAE (*n* = 250) and Bahrain (*n* = 170) (total *N* = 613). Participants selected preferred colours for scrubs and multiple dental items (PPE, chairside disposables, dental chair and wall colour), indicated scrub colour by clinical scenario (routine, surgical, paediatric), endorsed ‘comfortable’ colours (select-all), and rated five attitudinal statements on a 5-point Likert scale. Between-country differences were examined using chi-square tests (Cramer’s *V*) and Kruskal–Wallis tests; additional modelling included GEE for comfort endorsements and exploratory logistic regression for scenario switching.

**Results:**

Scrub preferences clustered around classic colours (blue/black), with modest between-country differences (*χ*² = 24.09, df = 10, *p* = 0.007; *V* = 0.14). Oman most preferred black (50.3%) then blue (41.5%); the UAE preferred blue (52.8%) then black (42.0%); Bahrain preferred blue (47.1%) then black (37.1%). Scenario framing increased openness to non-classic colours in paediatric care (red and yellow), and only 16.6% retained the same scrub colour across all scenarios. For other items, classic/neutral tones predominated, with significant country differences for most categories (including masks, wall colour, dental chair, dental bib, disposable kit and suction tips), while glove colour distributions were broadly similar. Warm/yellow operatory lighting was preferred over white lighting overall (60.2%) with negligible between-country differences (*p* = 0.881). Comfort endorsements were highest for blue and white; patterned prints showed the clearest country separation (UAE 50.4% vs Oman 36.8% and Bahrain 35.9%), persisting in adjusted GEE models. Most respondents disagreed that darker colours appear less hygienic, although distributions differed modestly across countries (Kruskal–Wallis *p* = 0.010).

**Conclusions:**

Colour preferences in GCC dental settings are anchored in a stable ‘classic’ palette, while scenario and item context introduce actionable departures, particularly for paediatric care. Black competed directly with blue across several domains and was not generally viewed as less hygienic, supporting its inclusion within a ‘safe’ procurement palette. Preference for warm lighting suggests a practical environmental lever to enhance perceived comfort.

## Introduction

Colour is a powerful nonverbal cue in healthcare environments, shaping patient perceptions of trust, professionalism, competence, hygiene, anxiety and comfort. The choice of colour in dental and medical attire, personal protective equipment (PPE) and operatory environments is not merely aesthetic; it can influence psychological outcomes, patient cooperation and even physiological stress responses [1].

Scrubs are widely accepted in contexts where practicality and perceived hygiene are prioritised, notably surgical and emergency settings, where they are associated with cleanliness, efficiency and competence [2, 3]. Blue and green scrubs are frequently linked to calmness and trust, whereas black scrubs may attract less favourable judgements in some populations [4, 5]. At the other end of the spectrum, casual attire typically receives the lowest preference ratings and may be interpreted as less professional or less knowledgeable, although some evidence suggests that casual dress can reduce psychological distance and ‘humanise’ clinicians in certain inpatient contexts [6].

Preferences are not uniform and appear to be moderated by demographic and cultural context. Older patients and those from lower socioeconomic backgrounds more often prefer traditional formal attire and white coats, associating these with authority and reliability [5, 7]. Younger patients tend to be more accepting of scrubs or more casual dress, potentially reflecting changing social norms and media portrayals of healthcare professionals [7, 8]. Cross-cultural work further indicates that modest and professional appearance may be especially emphasised in Middle Eastern and South Asian settings, whereas preferences in Western contexts can vary by ethnicity and clinical situation [9, 10]. These patterns are highly relevant to dental care because dentistry involves close interpersonal distance and prolonged face-to-face interaction, where visual cues may carry additional weight in shaping comfort and cooperation.

Gender may also shape how attire is interpreted, although findings are mixed. Some studies suggest that female clinicians face stricter appearance expectations and may be judged more harshly on dress-related cues, while others report minimal gender effects [11]. In dental contexts specifically, there is evidence that patients may hold more detailed expectations about grooming, such as a preference for tied-up hair for female dentists and for male dentists to be clean-shaven [8]. Such findings reinforce the idea that ‘professional appearance’ is not a single construct but a composite of attire style, colour and grooming cues.

At the same time, infection-control considerations have introduced tension between traditional symbols of professionalism and hygienic practice. Despite documented contamination risks associated with white coats, many patients continue to prefer them due to their symbolic association with credibility and trust [3, 12, 13]. Protective equipment, including face masks, gloves and head caps, generally receives strong support, particularly among younger cohorts, whereas items such as safety glasses may be less consistently endorsed, possibly due to limited public familiarity [8, 14]. Beyond attire itself, the visual environment may also influence patient comfort and perceived quality of care. Clinic colour schemes and surrounding cues can contribute to emotional appraisal; neutral or light tones are commonly described as calming and may reduce anxiety and support confidence in healthcare settings [8].

Collectively, existing evidence indicates that attire functions as a rapid visual heuristic through which patients form expectations about professionalism, competence, hygiene and approachability, with colour adding an additional layer that can shape emotional responses such as calmness or anxiety [4, 5, 8, 15]. This is particularly pertinent to dentistry, where close interpersonal distance, sustained chairside interaction and the salience of protective equipment and instruments may amplify the influence of appearance-related cues, including the colours of scrubs, masks, gloves and operatory items [8, 14]. However, prior work has largely centred on patient impressions within clinical encounters and typically emphasises broad ‘traditional’ preferences such as white coats and conventional scrub colours [13]. Fewer studies extend this lens to a wider set of dental items and environmental elements in ways that can inform practical design and procurement decisions, particularly within GCC contexts where modesty and professional appearance cues may be weighted differently [9, 10].

Exploring these perceptions among non-healthcare university students is informative because this group represents a large, educated segment of current and future service users, and their expectations may reflect evolving social norms and media influences [7, 8]. Therefore, this study investigated colour preferences for dental attire and selected clinical and operatory items, and examined their relationship with perceived comfort and key attitudinal constructs (trust, hygiene and anxiety), comparing responses across Oman, the UAE and Bahrain.

## Methods

### Study design and setting

This cross-sectional, web-based survey is reported with reference to the Strengthening the Reporting of Observational Studies in Epidemiology (STROBE) statement for observational studies [16]. The study was conducted in three Gulf Cooperation Council countries, namely Oman, the United Arab Emirates, and Bahrain, to examine colour preferences for dental attire and selected clinical and operatory items, and to explore related perceptions of comfort, professionalism, trust, hygiene, and anxiety.

#### Participants and recruitment

The target population comprised non-healthcare university students, defined as students not enroled in dentistry, medicine, nursing, or allied health programmes. Eligibility was confirmed using a screening item within the questionnaire. Participants were recruited using a convenience approach through university-linked distribution channels, including student mailing lists and course announcements, as applicable in each country. The questionnaire was administered electronically in a web-based format, and participation was voluntary and anonymous, with electronic informed consent obtained at the start of the survey. This population was selected because it represents a large, educated segment of current and future service users whose expectations may reflect evolving social norms and media influences.

#### Questionnaire content and study variables

A structured questionnaire was used and included: (1) demographics, namely age group, gender, and dental-visit frequency, (2) single-choice colour preference items covering attire and clinical or operatory components, including scrubs, gown, scrub cap, mask, gloves, patient goggles, dentist goggles, rubber dam, instruments, disposable kit, operatory wall colour, plastic cup, impression tray, suction tips, dental bib, dental chair, hand mirror, and operatory lighting preference (warm/yellow versus white), (3) scenario-specific scrub preferences (routine care, surgical setting, paediatric setting), (4) a select-all-that-apply comfort item covering blue, green, white, black, yellow, red, pastel colours, and patterned prints, (5) exposure and rationale items, including colours previously seen for scrubs, the participant’s single top priority among pre-defined domains, and single-choice reason-for-choice items for scrubs, wall colour, and chair colour, and (6) five attitudinal statements rated on a 5-point Likert scale from strongly disagree to strongly agree. These statements addressed whether non-classic colours reduce trust, bright colours increase anxiety, darker colours look less hygienic, dental attire should be modest, and neutral colours are always preferable.

The primary descriptive outcomes were the distributions of preferred colours for scrubs overall and by scenario, and the distributions for other attire and operatory items, including lighting preference. Secondary outcomes included the proportion selecting each option in the comfort item, within-respondent switching across scenarios, and country differences in Likert responses and in the number of comfort options selected. For modelling, colours were grouped a priori using a pragmatic study-specific classification. ‘Classic’ colours were defined as those conventionally associated with healthcare or dental attire and commonly encountered in clinical settings (blue, black, white, and green), whereas ‘non-classic’ colours referred to less conventional or more context-dependent options (red, yellow, pastel colours, and patterned prints).

### Data collection procedure

Responses were collected anonymously through the web-based questionnaire, and participants provided electronic consent before proceeding to the survey items.

### Bias and study size

Several steps were taken to improve response quality and reduce potential bias. Restricting the sample to non-healthcare students was intended to reduce the influence of professional training on colour-related perceptions, while anonymous participation and standardised questionnaire wording were used to minimise social desirability bias and inconsistency in administration. The final sample size reflected the number of eligible respondents reached through the participating university-linked channels during the survey period.

### Statistical analysis

Analyses were conducted in R (v4.4.3). Categorical outcomes were summarised as counts and percentages with 95% confidence intervals for key proportions. Between-country differences in categorical outcomes were tested using chi-square tests of independence, with Cramer’s *V* reported as an effect size. For selected contrasts, absolute percentage-point differences between countries were reported with 95% confidence intervals.

Likert items were treated as ordinal outcomes and summarised descriptively using mean and median values. Responses were coded as 1 for strongly disagree, 2 for disagree, 3 for neutral, 4 for agree, and 5 for strongly agree. Between-country comparisons for Likert items used Kruskal–Wallis tests, with *H* and epsilon-squared reported as effect sizes. Where an overall Kruskal–Wallis test was significant, pairwise Mann–Whitney *U* tests were conducted with Holm correction for multiple testing, and rank-biserial correlations were reported as effect sizes.

For the select-all comfort item, each option was coded as a binary variable indicating whether it was selected or not selected. Country differences in comfort selections were additionally examined using generalised estimating equations with a binomial distribution and logit link, clustering by Respondent ID to account for within-respondent correlation across comfort options. Adjusted predicted probabilities were generated by country while holding demographic covariates at their modal categories. An exploratory adjusted logistic regression model was fitted to examine predictors of preference switching. Item non-response was handled using available-case analysis for each test, with no imputation. Statistical significance was set at a two-sided alpha of 0.05. Sensitivity analyses included complete-case analyses and restriction to respondents reporting regular dental attendance, defined as every 6 months or once a year, with repetition of the corresponding between-country tests.

### Ethical considerations

Ethical approval was obtained from the institutional review board (approval number REC-26010101F). All participants provided electronic informed consent prior to participation.

## Results

A total of 613 respondents from three GCC countries completed the survey (Oman *n* = 193; UAE *n* = 250; Bahrain *n* = 170). Missing data were minimal (maximum item-level missingness 0.7%). Participant characteristics were broadly similar across countries (gender, age group and dental-visit frequency; all *p* > 0.89).

### Preferred scrub colour (overall)

**Fig. 1:**
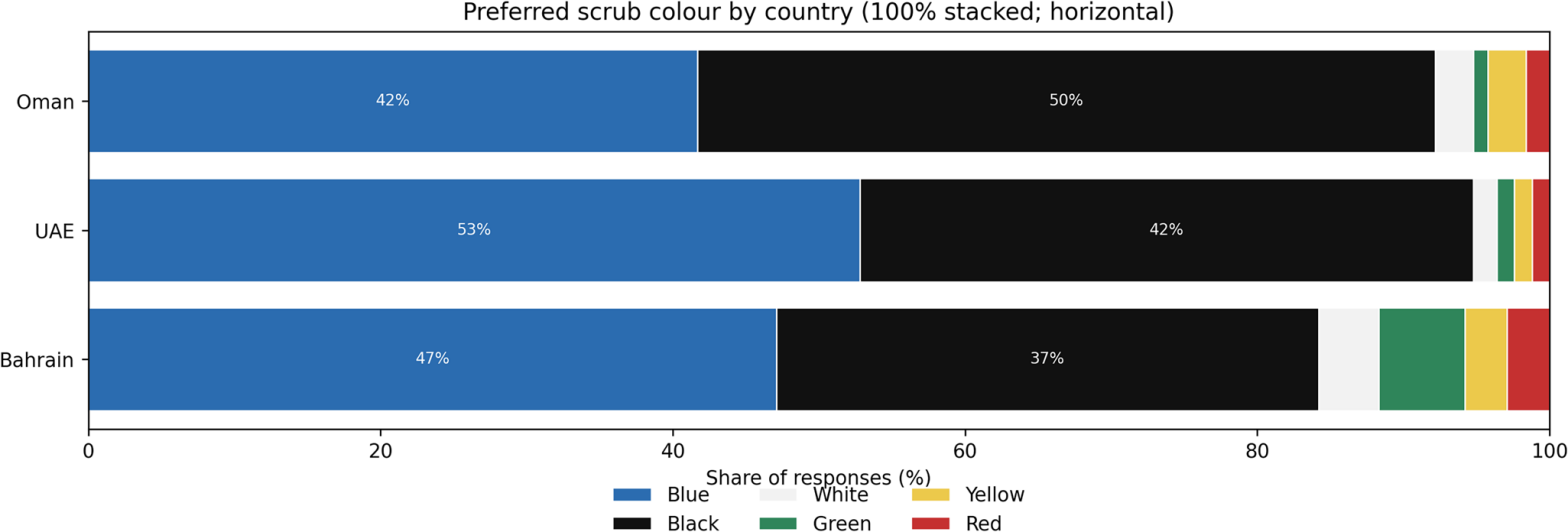
Distribution of respondents’ single best choice of scrub colour in Oman (*n* = 193), the UAE (*n* = 250) and Bahrain (*n* = 170). Bars show the share of responses (%) for each colour, standardised to 100% within each country. Blue and black dominated in all three settings, with Oman showing a higher black preference and the UAE and Bahrain showing a higher blue preference.

Across countries, preferences clustered around classic scrub colours, with blue and black dominating (Fig. 1). Country differences were present but modest (*χ*² = 24.09, df = 10, *p* = 0.007; Cramer’s *V* = 0.14). In Oman, black was most preferred (50.3%, 95% CI: 43.3–57.2) followed by blue (41.5%, 95% CI: 34.7–48.5). In the UAE, blue was most preferred (52.8%, 95% CI: 46.6–58.9) with black second (42.0%, 95% CI: 36.0–48.2). In Bahrain, blue was most preferred (47.1%, 95% CI: 39.7–54.5) with black second (37.1%, 95% CI: 30.2–44.5). The absolute difference in blue preference between the UAE and Oman was +11.3 percentage points (95% CI: +2.0 to +20.4).

### Scenario-specific scrub colour and within-respondent switching

**Fig. 2:**
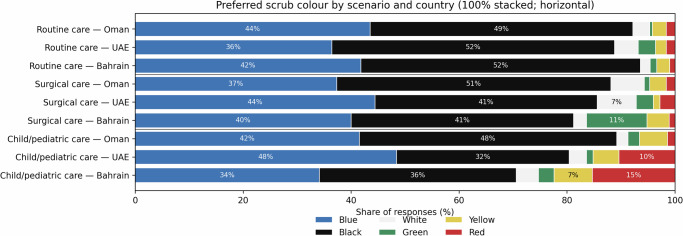
Respondents’ preferred scrub colour by clinical scenario (routine care, surgical care, child/paediatric care) across Oman, the UAE and Bahrain. Bars show the share of responses (%) for each colour, standardised to 100% within each scenario-country combination. Scenario framing altered preferences, with increased selection of non-classic colours in the paediatric scenario, while routine and surgical contexts remained anchored in classic colours.

Scenario framing altered scrub preferences (Fig. 2). For routine care, black was most selected in all three countries (Oman 48.7%, UAE 52.4%, Bahrain 51.8%). For surgical care, green increased notably in Bahrain (11.2%) compared with Oman (1.0%) and the UAE (3.2%). For child/paediatric care, non-classic colours increased: red rose to 10.4% in the UAE and 15.3% in Bahrain (Oman 1.6%), with a parallel increase in yellow (Oman 5.2%, UAE 4.8%, Bahrain 7.1%). Overall, only 16.6% of respondents retained the same scrub colour across all three scenarios, indicating that contextual cues influenced preferences.

In exploratory adjusted logistic regression modelling switching to a non-classic colour in the paediatric scenario (vs retaining classic colours), country remained the primary predictor: compared with Bahrain, the odds of switching were lower in Oman (OR: 0.25, 95% CI: 0.12–0.50, *p* < 0.001), while the UAE showed a similar direction that did not reach conventional significance (OR: 0.62, 95% CI: 0.37–1.04, *p* = 0.072). Attitudinal items and demographics were not independently associated with switching in this model (all *p* ≥ 0.25).

### Colour preferences for other items (PPE, equipment, and environment)

**Fig. 3:**
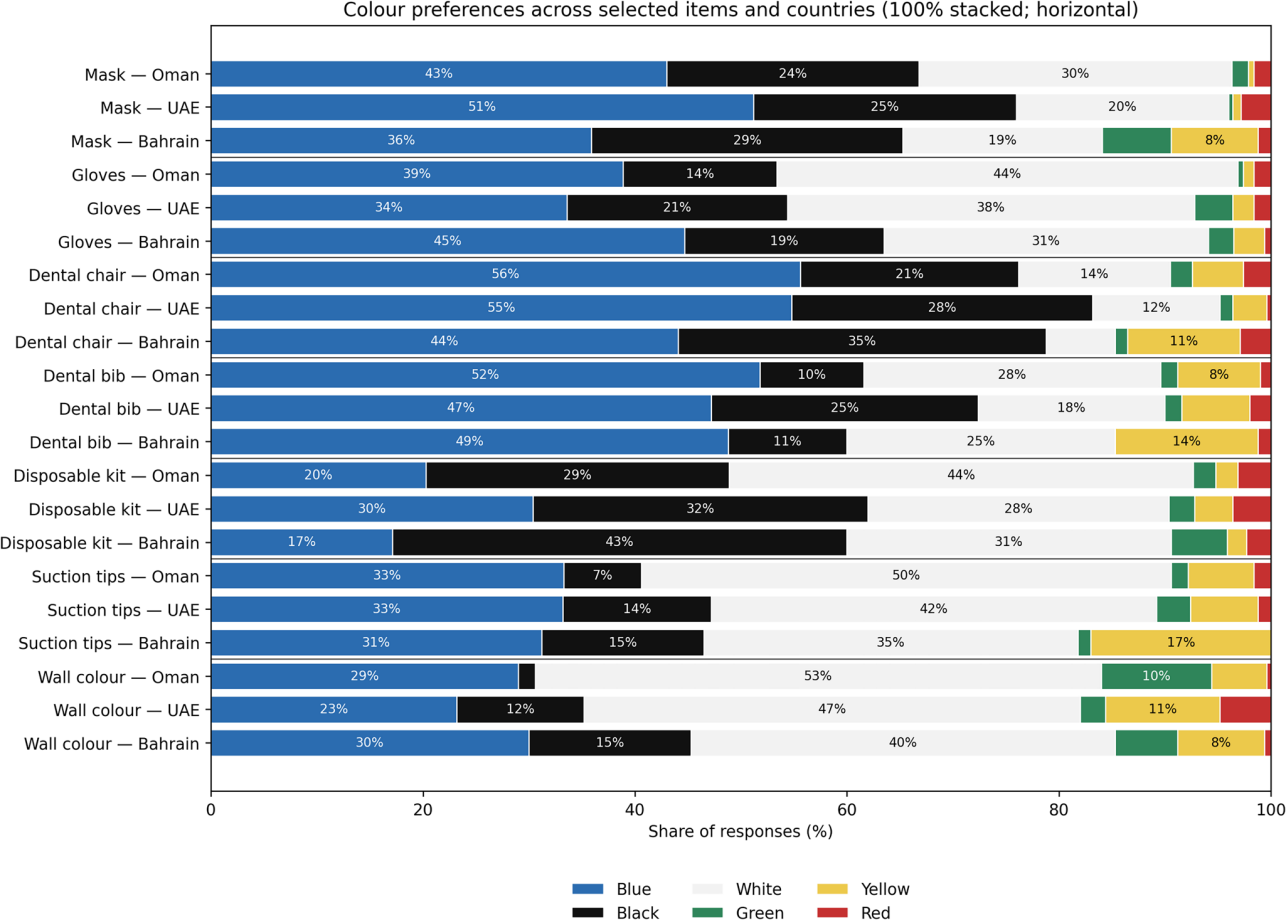
Colour preferences for selected non-attire items (mask, gloves, dental chair, dental bib, disposable kit, suction tips, and operatory wall colour) in Oman, the UAE and Bahrain. Bars show the share of responses (%) for each colour, standardised to 100% within each item-country combination. Preferences generally favoured classic or neutral colours, with item- and country-specific dispersion patterns, particularly for smaller disposables and environmental elements.

Item-level preferences also favoured classic palettes, but with country-specific nuances (Fig. 3). Between-country differences were statistically significant for most items, including mask (*χ*² = 55.71, df = 10, *p* < 0.001; Cramer’s *V* = 0.213), wall colour (*χ*² = 53.44, df = 10, *p* < 0.001; *V* = 0.209), dental chair (*χ*² = 29.99, df = 10, *p* < 0.001; *V* = 0.157), dental bib (*χ*² = 35.41, df = 10, *p* < 0.001; *V* = 0.170), disposable kit (*χ*² = 29.03, df = 10, *p* = 0.001; *V* = 0.154), and suction tips (*χ*² = 30.52, df = 10, *p* < 0.001; *V* = 0.158). In contrast, glove colour preferences did not reach conventional significance (*χ*² = 16.85, df = 10, *p* = 0.078; *V* = 0.117), indicating broadly similar distributions across countries.

For masks, blue was the modal preference in all three countries, but with clear variation in magnitude: UAE 51.2% (95% CI: 45.0–57.3), Oman 43.0% (36.2–50.1), and Bahrain 35.9% (29.1–43.3). The UAE-Bahrain difference for selecting blue masks was +15.3 percentage points (95% CI: +5.6 to +24.5). Bahrain’s mask preferences were more dispersed, with relatively higher selection of black (29.4%, 23.1–36.7) and yellow (8.2%, 5.0–13.3).

For wall colour, white was the leading preference in all countries but declined from Oman to Bahrain: Oman 53.4% (46.3-60.3), UAE 46.8% (40.7–53.0), and Bahrain 40.0% (32.9–47.5). Preference for black walls was higher in Bahrain (15.3%, 10.7–21.5) and the UAE (12.0%, 8.5–16.6) than in Oman (1.6%, 0.5–4.5), with the Bahrain-Oman difference +13.7 percentage points (95% CI: +8.3 to +20.0).

For lighting preference, respondents consistently preferred warm/yellow lighting over white lighting (overall 60.2%, 95% CI: 56.3–64.0). This pattern was similar in each country: Oman 61.7% (95% CI: 54.6–68.2), UAE 59.6% (95% CI: 53.4–65.5) and Bahrain 59.4% (95% CI: 51.9–66.5). Between-country differences were negligible (*χ*² = 0.253, df = 2, *p* = 0.881; Cramer’s *V* = 0.02), indicating a broadly shared preference for warmer operatory lighting.

For the dental chair, blue dominated in Oman (55.6%, 48.4–62.5) and the UAE (54.8%, 48.6–60.9). Bahrain remained blue-led (44.1%, 36.9–51.6) but showed a higher preference for black chairs (34.7%, 28.0–42.1) compared with Oman (20.6%, 15.5–27.0), a difference of +14.1 percentage points (95% CI: +4.8 to +23.1).

For the dental bib, blue was consistently the top choice (Oman 51.8%, 44.8–58.8; UAE 47.2%, 41.1–53.4; Bahrain 48.8%, 41.4–56.3). The UAE showed a higher selection of black bibs (25.2%, 20.2–30.9) than Oman (9.8%, 6.4–14.9), an absolute difference of +15.4 percentage points (95% CI: +8.3 to +22.0).

For the disposable kit, Oman preferred white (43.8%, 36.9–50.8), whereas Bahrain showed a higher preference for black (42.9%, 35.7–50.5). The absolute difference in selecting black for the disposable kit between Bahrain and Oman was +14.3 percentage points (95% CI: +4.4 to +23.9). The UAE was more evenly distributed across black (31.6%, 26.2–37.6), blue (30.4%, 25.0–36.4), and white (28.4%, 23.2–34.3), indicating greater tolerance for multi-colour options in this category.

For suction tips, white was the leading preference but again showed a gradient across countries: Oman 50.0% (43.0–57.0), UAE 42.0% (36.0–48.2), and Bahrain 35.3% (28.5–42.7). Bahrain showed a higher preference for yellow suction tips (17.1%, 12.1–23.4) than Oman (6.3%, 3.6–10.6), a difference of +10.8 percentage points (95% CI: +4.2 to +17.7).

### Colours perceived as comfortable (select-all-that-apply)

**Fig. 4:**
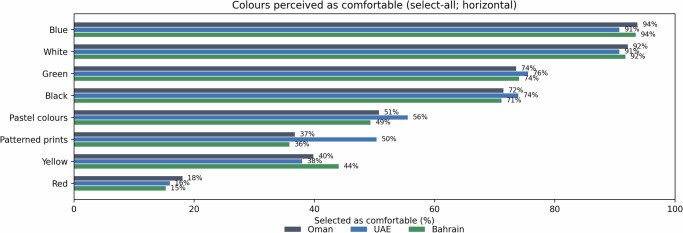
Proportion of respondents selecting each colour as ‘comfortable’ in a select-all-that-apply item, shown separately for Oman, the UAE and Bahrain. Values represent endorsement rates (%) rather than a single best choice, allowing multiple selections per respondent. Blue and white were most frequently endorsed as comfortable across all countries; patterned prints showed the clearest between-country separation, with higher endorsement in the UAE.

Respondents were allowed to endorse multiple colours as ‘comfortable’ (Fig. 4). Comfort endorsements were highest for blue (overall 92.5%) and white (91.5%), followed by green (74.6%) and black (72.4%). Patterned prints showed the clearest country separation, endorsed by 50.4% in the UAE (95% CI: 44.2–56.5) versus 36.8% in Oman (95% CI: 30.3–43.8) and 35.9% in Bahrain (95% CI: 29.1–43.3) (*χ*² = 12.00, df = 2, *p* = 0.002; Cramer’s *V* = 0.140). The UAE-Oman difference for patterned prints was +13.6 percentage points (95% CI: +4.3 to +22.5), and the UAE-Bahrain difference was +14.5 percentage points (95% CI: +4.9 to +23.7). The mean number of ‘comfortable’ colours selected was similar across countries (Oman 4.77 ± 1.26, UAE 4.91 ± 1.20, Bahrain 4.75 ± 1.23; medians all 5).

In adjusted GEE models accounting for within-respondent correlation and controlling for demographics, the UAE remained more likely to endorse patterned prints as comfortable than Oman (OR: 1.75, 95% CI: 1.19–2.57) and Bahrain (OR: 1.82, 95% CI: 1.22–2.71); adjusted predicted probabilities were 35.1, 48.7, and 34.2%, respectively.

### Attitudinal items (Likert responses)

Attitudinal items showed strong agreement that classic/neutral presentation matters. Overall, respondents agreed that non-classic colours reduce trust and that bright colours may increase anxiety, and strongly agreed that attire should be modest and that neutral colours are preferable; these items did not differ meaningfully between countries (Kruskal–Wallis *p* values > 0.56).

In contrast, perceptions of hygiene related to darker colours showed a distinct pattern. Overall, respondents disagreed with the statement that darker colours appear less hygienic. Disagreement predominated in all countries (Oman 87.0%, 95% CI: 81.6–91.1; UAE 77.2%, 95% CI: 71.6–82.0; Bahrain 86.5%, 95% CI: 80.5–90.8), but distributions differed across countries (Kruskal–Wallis *H* = 9.21, *p* = 0.010; *ε*² = 0.012, small effect). The UAE showed a higher minority agreement (agree/strongly agree 13.2%, 95% CI: 9.6–18.0) than Oman (5.7%, 3.2–9.9) and Bahrain (4.1%, 2.0–8.3). Post hoc Mann–Whitney comparisons with Holm correction indicated that responses in the UAE were shifted towards higher agreement compared with Bahrain (*p*Holm = 0.0157; rank-biserial *r* = 0.146), whereas the Oman-UAE contrast did not remain significant after correction (*p*Holm = 0.059).

### Sensitivity checks

Results were unchanged in complete-case analyses. When restricting analyses to respondents reporting regular attendance (every 6 months/once a year), country differences remained for scrub preference (*χ*² *p* = 0.0177; *V* = 0.197) and patterned-print comfort endorsements (*χ*² *p* = 0.00146; *V* = 0.217), while the between-country difference for the ‘dark less hygienic’ item attenuated (Kruskal–Wallis *p* = 0.162); however, the UAE continued to show the highest minority agreement in this restricted subset. Key between-country contrasts are shown in Table 1.

**Table 1:** Selected between-country contrasts with 95% CIs and corresponding test statistics.

Outcome	Oman	UAE	Bahrain	Between-country comparison
Scrubs: blue preference	41.5% (34.7–48.5)	52.8% (46.6–58.9)	47.1% (39.7–54.5)	*χ*² *p* = 0.007 (*V* = 0.140); UAE-Oman +11.3 pp (95% CI: +2.0 to +20.4)
Comfort: patterned prints	36.8% (30.3–43.8)	50.4% (44.2–56.5)	35.9% (29.1–43.3)	*χ*² *p* = 0.002 (*V* = 0.140); UAE-Oman +13.6 pp (95% CI: +4.3 to +22.5)
‘*Dark less hygienic*’: disagree/strongly disagree	87.0% (81.6–91.1)	77.2% (71.6–82.0)	86.5% (80.5–90.8)	Kruskal–Wallis *p* = 0.010 (*ε*² = 0.012); UAE vs Bahrain post hoc *p*Holm = 0.0157
Disposable kit: black preference	28.5% (23.0–35.2)	31.6% (26.2–37.6)	42.9% (35.7–50.5)	*χ*² *p* = 0.001 (*V* = 0.154); Bahrain-Oman +14.3 pp (95% CI: +4.4 to +23.9)

*pp* percentage points, *CI* confidence interval, *V* Cramer’s *V*, *ε*^*²*^ epsilon-squared, *pHolm* Holm-adjusted *p* value.

## Discussion

Across three GCC countries, colour preferences for dental scrubs and chairside and operatory items clustered strongly around a ‘classic’ palette (blue, black, white, green), while also showing context- and country-specific departures with practical relevance. The most consistent pattern was that blue and black dominated scrub preferences overall, with only modest between-country differences. However, scenario framing produced clearer shifts: paediatric dentistry elicited greater openness to non-classic colours (notably red and yellow), whereas routine and surgical contexts remained anchored in classic tones. This pattern supports the interpretation that colour is not simply an aesthetic choice but a cue that is ‘read’ differently depending on perceived risk, formality and the emotional tone of the clinical encounter [17–23].

A potentially distinctive contribution of this work is that black was not merely acceptable; it competed directly with blue and, in some contexts (e.g. routine-care scrubs), was the modal choice. This is notable because patient-perception studies in other settings have reported that darker attire, and black in particular, may be interpreted less favourably, being linked to more negative trait attributions and harsher clinical judgements in experimental vignettes [4]. In the present data, this ‘black penalty’ was not observed at the attitudinal level: the majority in all countries disagreed that darker colours appear less hygienic, with between-country differences driven by a higher minority agreement in the UAE rather than a reversal of the overall direction. These findings suggest that colour-meaning associations are culturally and temporally contingent, and that what is considered ‘non-classic’ or ‘risk-signalling’ in one population may not generalise to another [1, 24, 25]. Practically, this implies that black can plausibly be treated as part of a ‘safe’ procurement palette in GCC settings, particularly for professional attire and several non-attire items, rather than as an inherently polarising option.

The paediatric scenario produced the most visible relaxation of colour conservatism, with increased selection of red and yellow relative to routine and surgical scenarios. This direction aligns with a broader evidence base suggesting that child-friendly attire and colourful clinic cues may reduce anxiety and improve cooperation in children [19, 21, 26, 27]. The same respondents often changed preferences across scenarios (low within-respondent consistency), reinforcing that these are not fixed personal tastes but context-conditioned judgements. The adjusted model outputs (country-predicted switching probabilities) support this interpretation: country was a stronger predictor of switching than attitudinal items, implying that shared social norms and exposure may shape what feels acceptable in paediatric dentistry more than individual beliefs captured by the Likert items.

For most non-attire items, preferences still favoured classic or neutral tones, but with ‘country signatures’ that are potentially helpful for real-world design decisions (e.g. Bahrain showing higher tolerance for black in certain categories such as the disposable kit, and more dispersed distributions for smaller disposables such as suction tips). This matters because much of the published ‘attire’ literature focuses narrowly on coats and scrubs rather than the full visual field of a dental visit [28, 29]. Dentistry is unusually cue-rich: patients are exposed simultaneously to PPE, instruments and disposables, the dental chair, walls and lighting; therefore, the ‘colour experience’ is composite rather than single-item.

The finding that a majority in all three GCC countries preferred warm or yellow lighting over white lighting is consistent with the broader clinical-environment literature, which generally reports higher perceived comfort for warm or warm-neutral white illumination (3000–4500 K) and lower comfort ratings for cooler, blue-enriched ‘clinical’ lighting (>5500 K) [30–32]. Warm light has been linked to subjective relaxation and reduced visual harshness, whereas cooler light may support alertness and task performance but can be experienced as less comfortable; preferences may also vary by demographic factors, and the evidence base remains stronger for self-reported comfort and satisfaction than for acute physiological outcomes in real clinical settings [33, 34]. Lighting temperature appears to be a modifiable environmental lever that may enhance perceived comfort without altering core clinical protocols (e.g. warmer settings for patient-facing time and ambience, with the option of cooler or brighter task lighting when required for clinical visibility) [35]. Consistent with this, warm (3000 K) illumination has been associated with a more attractive ambience in consumer settings [36], and experimental indoor-environment research indicates that very cool, high-CCT light (e.g. 5800 K) may be perceived as less visually comfortable under cooler thermal conditions, with visual comfort correlating with thermal comfort [37].

The ‘select-all-that-apply’ comfort item adds an important layer beyond single best-choice preferences and helps avoid an overly reductive conclusion that classic colours dominate. Colours that are not the top preference may nonetheless be regarded as acceptable or comfortable by a substantial minority. In this context, the additional modelling was informative: the GEE analyses identified patterned prints as the clearest between-country separator, with higher endorsement in the UAE, while most other comfort options showed broadly similar adjusted probabilities. This pattern supports a practical interpretation in which a core palette can be standardised regionally, while selective customisation (e.g. patterned prints in paediatric settings or for specific staff groups) can be implemented where tolerance is demonstrably higher, without assuming that such acceptability generalises across neighbouring countries.

Two recurring findings in the attire literature are a preference for more formal or professional dress and the strong moderating role of context, including speciality, setting and patient expectations [14, 38]. The present findings are broadly consistent with a professional heuristic, with classic colours predominating and strong endorsement of modesty and neutral presentation. At the same time, the results challenge an often-assumed hygiene narrative in which darker colours are viewed as less hygienic. The contrast with experimental evidence in which black scrubs were penalised is therefore informative [4], suggesting that colour meanings may shift with region, time and local exposure to healthcare branding and uniform norms. This interpretation is also consistent with the view that attire is only one component of patients’ judgements and may be outweighed by other cues once a baseline of professionalism is established [1, 6, 24, 25]. One possible explanation for the acceptability of darker palettes is broader aesthetic normalisation in younger cohorts, who are increasingly exposed to ‘dark mode’ interfaces in everyday digital use [39]. Prior work on background-mode preferences notes that younger generations may favour dark mode more than older adults, who tend to prefer light mode, although evidence is mixed across applications and contexts [39].

From a service-design perspective, the results support a tiered approach: maintain classic colours as the default for routine and surgical dentistry, allow controlled expansion of non-classic options for paediatric care, and consider country-specific procurement choices for high-visibility items (chairs and walls) and frequently handled disposables. Given the consistent preference for warm lighting, clinics may also consider lighting temperature as a low-disruption lever to improve perceived comfort without major changes to uniforms or equipment.

From a manufacturer and procurement perspective, these findings support evidence-based portfolio design rather than relying on simple ‘most popular colour’ rankings. The distributions indicate a robust GCC ‘safe’ palette across scrubs, PPE and operatory items, while also identifying a small number of context- or country-specific departures that can be offered as targeted add-ons; this approach is consistent with product-line evidence showing that optimal colour offerings should account for preference heterogeneity and substitution trade-offs when deciding how many options to carry [40]. Moreover, consumer research on colour combinations suggests that users tend to prefer visually coherent schemes (identical or closely related hues) and often construct designs using a limited palette, which reduces complexity and avoids unnecessary proliferation of options [41]. In practical terms, suppliers could prioritise coordinated bundles based on the core palette (including black as a viable ‘classic’ option in GCC settings) and reserve a small set of accent colours or patterned variants for specific use-cases such as paediatric dentistry or markets demonstrating higher tolerance, thereby improving acceptability while keeping Stock Keeping Unit complexity manageable.

Several questions remain unanswered and should guide future research. First, these findings were based on non-healthcare university students rather than patients observed during actual dental encounters, so it remains unclear how strongly stated preferences translate into real-time behaviour, anxiety, cooperation, or trust in clinical settings. Second, although the paediatric scenario produced greater openness to non-classic colours, this inference was drawn from adult respondents imagining paediatric contexts rather than from children or parents themselves. Future studies should therefore include adult dental patients, paediatric patients and parents, and should test colour preferences under more ecologically valid conditions, ideally using photographs, simulated clinics, or real clinical environments. Experimental studies could also examine whether modifying attire colour, operatory colour schemes, or lighting temperature produces measurable changes in anxiety, perceived professionalism, satisfaction, or treatment cooperation. Finally, broader cross-cultural work is needed to determine whether the apparent acceptability of black and the preference for warm lighting observed here are stable across age groups, care settings, and GCC populations, or whether they reflect cohort effects, local exposure patterns, and changing visual norms.

Strengths include the multi-country GCC design, the breadth of items assessed beyond attire, scenario-based testing, and the use of both single best-choice preferences and acceptability endorsements. Limitations include convenience sampling and reliance on self-reported preferences rather than observed behaviour; responses may also have been shaped by how participants envisaged the scenarios and by unmeasured prior exposure to local uniform norms. Isolating colour effects from lighting and other environmental cues is inherently challenging, as also noted in related literature [35, 42]. In addition, the greater openness to non-classic colours in the paediatric scenario reflects adult respondents’ views about a paediatric context rather than direct evidence from children or parents, and should therefore be interpreted cautiously. These limitations indicate the need for future patient-based and experimentally grounded studies that test whether the preferences identified here translate into observable effects in real clinical practice.

## Conclusions

In this three-country GCC survey, colour preferences for dental scrubs, PPE and operatory elements were strongly anchored to a classic palette (blue, black, white and green), with only modest between-country differences for most items. However, preferences were clearly scenario-dependent: paediatric dentistry elicited greater tolerance for non-classic colours, and within-respondent switching across scenarios was common, indicating context-conditioned judgements rather than fixed personal tastes. Notably, black competed directly with blue as a leading scrub choice and was not associated with a dominant perception that darker colours appear less hygienic. Warm/yellow operatory lighting was consistently preferred across all countries, suggesting a low-disruption environmental lever to enhance perceived comfort. Collectively, these findings support a tiered approach to clinical design and procurement in GCC settings: standardise a core ‘safe’ palette for routine and surgical care, allow controlled expansion for paediatric contexts, and consider lighting temperature and selected country-specific preferences when designing patient-facing environments.

## Data Availability

All data generated or analysed during this study are included in this published article. Additional details, if required, are available from the corresponding author upon reasonable request.

## References

[1] Shoyama S, Hisae AOKI, Kubota K, Shimokita H, Tochihara Y. Impressions and assessment of medical uniforms in different colors. J Japan Res Assoc Text End Uses. 2014;55:16.

[2] Jennings JD, Pinninti A, Kakalecik J, Ramsey FV, Haydel C. Orthopaedic physician attire influences patient perceptions in an urban inpatient setting. Clin Orthop Relat Res. 2019;477:2048–58. 31294719 10.1097/CORR.0000000000000822 PMC7000081

[3] Kurihara H, Maeno T, Maeno T. Importance of physicians’ attire: factors influencing the impression it makes on patients, a cross-sectional study. Asia Pac Fam Med. 2014;13:2. 24397871 10.1186/1447-056X-13-2 PMC3890493

[4] Hribar C, Chandran A, Piazza M, Quinsey C. Association between patient perception of surgeons and colour of scrub attire. JAMA Surg. 2023;158:422. 10.1001/jamasurg.2022.5837 PMC9857704 36630142

[5] Shimizu A, et al. Physician attire influences patient and family perceptions of care in the palliative care unit in Japan. Am J Hosp Palliat Med. 2022;39:907–12. 10.1177/10499091211051670 34706586

[6] Gherardi G, Cameron J, West A, Crossley M. Are we dressed to impress? A descriptive survey assessing patients’ preference of doctors’ attire in the hospital setting. Royal College of Physicians. 2009;9:519–24. 10.7861/clinmedicine.9-6-519 PMC4952286 20095290

[7] Van Blarcom J. Physician attire: a scholarly look. Hosp Pediatr. 2012;2:249–52. 24313034 10.1542/hpeds.2012-0052

[8] Pandey J, Kaur H, Izhar A, Batra P. Patient preference for dental clinical attire, hairdo, and infection control measures: a cross-sectional survey. Int J Curr Res Rev. 2020;12:1–9.

[9] Alsaeed S, Alghurairi N, Almutairi L, Alossimi A, Bin Fadhl A, Abahussain S. Factors that affect Saudi population preferences toward their dentist. Patient Prefer Adherence. 2021;15:2693–701. 34880604 10.2147/PPA.S333994 PMC8648270

[10] Ashraf MA, Yasser F, Azhar SM, Mobin A, Ashraf J. Patient’s perception about dental professionals attire. Pak J Med Health Sci. 2020;14:872–3.

[11] Payne RM, Tenenbaum MM. Commentary on: dress to impress: public perception of plastic surgeon attire. Aesthet Surg J. 2021;42:707–8. 10.1093/asj/sjab423 34932784

[12] Goyal S, Khot SC, Ramachandran V, Shah KP, Musher DM. Bacterial contamination of medical providers’ white coats and surgical scrubs: a systematic review. Am J Infect Control. 2019;47:994–1001. 30850250 10.1016/j.ajic.2019.01.012

[13] Tsagkaris C, Rueger M, Tschudi SB, Dreher T. White coats at a crossroads: hygiene, infection risk, and patient trust in healthcare attire—an umbrella review with quantitative synthesis and stress, weaknesses, opportunities, and threats analysis. Microorganisms. 2024;12:2659. 39770860 10.3390/microorganisms12122659 PMC11728839

[14] McKenna G, Lillywhite GRR, Maini N. Patient preferences for dental clinical attire: a cross-sectional survey in a dental hospital. Br Dent J. 2007;203:681–5. 18084213 10.1038/bdj.2007.1109

[15] Petrilli CM, Mack M, Petrilli JJ, Hickner A, Saint S, Chopra V. Understanding the role of physician attire on patient perceptions: a systematic review. BMJ Open. 2015;5:e006578. 25600254 10.1136/bmjopen-2014-006578 PMC4312788

[16] Von Elm E, Altman DG, Egger M, Pocock SJ, Gøtzsche PC, Vandenbroucke JP. The Strengthening the Reporting of Observational Studies in Epidemiology (STROBE) statement: guidelines for reporting observational studies. Lancet. 2007;370:1453–7. 18064739 10.1016/S0140-6736(07)61602-X

[17] Babaji P, Chauhan PP, Rathod V, Mhatre S, Paul U, Guram G. Evaluation of child preference for dentist attire and usage of camouflage syringe in reduction of anxiety. Eur J Dent. 2017;11:531–6. 29279683 10.4103/ejd.ejd_223_17 PMC5727742

[18] Babaji P, Chauhan P, Churasia VR, Kaur T, Singh S, Augustine M. A cross-sectional evaluation of children preference for dentist attire and syringe type in reduction of dental anxiety. Dent Res J. 2018;15:391–6. PMC6243804 30534166

[19] Bchara J, Abed D, Laflouf M, Massoud S, Alfeel J, Abed D. Child’s friendly dental attire, a game changer for anxiety and pain management in dental environment, a randomized clinical trial. Sci Rep. 2024;14:30182. 39632938 10.1038/s41598-024-81952-4 PMC11618609

[20] de Souza-Constantino AM, Conti ACDCF, Capelloza Filho L, Marta SN, de Almeida-Pedrin RR. Patients’ preferences regarding age, sex, and attire of orthodontists. Am J Orthod Dentofacial Orthop. 2018;154:829–34. 30477781 10.1016/j.ajodo.2018.02.013

[21] Kilic E, Aydinoglu S, Gunacar DN. White vs. colored coats: which reduces dental anxiety better? BMC Oral Health. 2025;25:661. 40301917 10.1186/s12903-025-05948-w PMC12042434

[22] Oliveira LB, Massignan C, De Carvalho RM, Savi MG, Bolan M, Porporatti AL, et al. Children’s perceptions of dentist’s attire and environment: a systematic review and meta-analysis. Int J Clin Pediatr Dent. 2020;13:700. 33976499 10.5005/jp-journals-10005-1839 PMC8060925

[23] Maganur PC, Vishwanathaiah S, Quadri MFA, Alsabi M, Modarba W, Aqeel W, et al. Color perception and its relation to dental anxiety in children. Dent Med Probl. 2024;61:671–7. 39451058 10.17219/dmp/145896

[24] Sadiq G, Sadiq S. The influence of physicians attire on patients perception in Rawalpindi/Islamabad, Pakistan. Rawal Med J. 2018;43:314.

[25] Umamaheshwari N, Asokan S, Kumaran TS. Child friendly colors in a pediatric dental practice. J Indian Soc Pedod Prev Dent. 2013;31:225–8. 24262394 10.4103/0970-4388.121817

[26] Singh V, Vashisth P, Naik S, Sharma S. Assessment of choice and anxiety toward different dentist’s attires among 5-12-year-old children in dental operatory. Indian J Dent Sci. 2023;15:180–5.

[27] Ravikumar D, Gurunathan D, Karthikeyan S, Subbramanian EMG, Samuel VA. Age and environment determined children’s preference towards dentist attire—a cross-sectional study. J Clin Diagn Res. 2016;10:ZC16. 27891450 10.7860/JCDR/2016/22566.8632 PMC5121796

[28] López-Tarruella J, Llinares Millan C, Serra Lluch J, Iñarra Abad S, Wijk H. Influence of color in a lactation room on users’ affective impressions and preferences. HERD. 2019;12:55–70. 30198330 10.1177/1937586718796593

[29] Kaja FJ, Asokan S, Pollachi-Ramakrishnan GP, Viswanath S, Thoppe-Dhamodharan YK. Impact of colors on dental anxiety in children: a scoping review. J South Asian Assoc Pediatr Dent. 2025;8:60–6.

[30] Bodrogi P, Guo X, Stojanovic D, Fischer S, Khanh TQ. Observer preference for perceived illumination chromaticity. Color Res Appl. 2018;43:506–16.

[31] Huang Z, Liu Q, Luo MR, Pointer MR, Wu B, Liu A. The whiteness of lighting and colour preference, Part 2: a meta-analysis of psychophysical data. Light Res Technol. 2020;52:23–35.

[32] Ronchi LR. Warm and cold light as related to fine grain of circadiancy. Light Eng. 2014;22:15–22.

[33] Gagne V, Turgeon R, Jomphe V, Demers CM, Hebert M. Evaluation of the effects of blue-enriched white light on cognitive performance, arousal, and overall appreciation of lighting. Front Public Health. 2024;12:1390614. 38813427 10.3389/fpubh.2024.1390614 PMC11133540

[34] Chen R, Tsai MC, Tsay YS. Effect of color temperature and illuminance on psychology, physiology, and productivity: an experimental study. Energies. 2022;15:4477.

[35] Hosseini SN, Walton JC, SheikhAnsari I, Kreidler N, Nelson RJ. An architectural solution to a biological problem: a systematic review of lighting designs in healthcare environments. Appl Sci. 2024;14:2945.

[36] Horská E, Berčík J. The influence of light on consumer behavior at the food market. J Food Prod Mark. 2014;20:429–40.

[37] Te Kulve M, Schlangen L, van Marken Lichtenbelt W. Interactions between the perception of light and temperature. Indoor Air. 2018;28:881–91. 30113746 10.1111/ina.12500

[38] Lill MM, Wilkinson TJ. Judging a book by its cover: descriptive survey of patients’ preferences for doctors’ appearance and mode of address. Br Med J. 2005;331:1524–7. 16373739 10.1136/bmj.331.7531.1524 PMC1322253

[39] Gazit T, Tager-Shafrir T, Zhong HX, Hung PC, Cheung V. The dark side of the interface: examining the influence of different background modes on cognitive performance. Ergonomics. 2025;68:1–14. 10.1080/00140139.2025.2483451 40131320

[40] Choi P, Orsborn S, Boatwright P. Bayesian analysis of color preferences: an application for product and product line design. Color Res Appl. 2016;41:445–56.

[41] Deng X, Hui SK, Hutchinson JW. Consumer preferences for color combinations: an empirical analysis of similarity-based color relationships. J Consum Psychol. 2010;20:476–84.

[42] Hoque S, Alcaraz M, Duncan A, McCarthy R. Lessons learned in the design of healthy indoor environments. In: 17th International Conference on Indoor Air Quality and Climate (INDOOR AIR 2022); 12-16 June 2022; Kuopio, Finland. Herndon, VA: International Society of Indoor Air Quality and Climate; 2022. p. 148.

